# Integrative analysis of Zidan Yinxie capsules for psoriasis treatment: Insights from network pharmacology, molecular dynamics simulation, and GEO database analysis

**DOI:** 10.1097/MD.0000000000044446

**Published:** 2025-09-26

**Authors:** Jianhua Bu, Caiqing Mao, Lisheng Nan, Xianwei Wu

**Affiliations:** a The First School of Clinical Medicine, Gansu University of Chinese Medicine, Lanzhou, Gansu, China; b Department of Dermatology, Gansu Provincial Hospital, Lanzhou, Gansu, China.

**Keywords:** molecular docking, molecular dynamics, psoriasis, traditional Chinese medicine, Zidan Yinxie capsules

## Abstract

The therapeutic efficacy of Zidan Yinxie capsules (ZDYXC) in psoriasis (PSO) has been clinically confirmed, yet its underlying molecular mechanisms remain unclear. We aimed to explore the mechanisms of ZDYXC in the treatment of PSO. To elucidate the mechanisms by which ZDYXC exerts its effects in PSO, active ingredients and their potential targets were first identified using the TCMSP database, while PSO-related genes were collected from several comprehensive databases, including GeneCards, Online Mendelian Inheritance in Man, Pharmacogenetics and Pharmacogenomics Knowledge Base, Therapeutic Target Database, and DrugBank. Common genes between ZDYXC targets and PSO-related genes were then screened, and an integrated network of these targets and active compounds was constructed with Cytoscape 3.10.1 software The Cytoscape Consortium; The Scripps Research Institute, La Jolla). Further analysis of protein–protein interactions among these intersection genes was performed using the STRING 12.0 platform (https://cn.string-db.org/), and hub targets were identified via the CytoNCA 2.1.6 plugin (The Cytoscape Consortium, The Scripps Research Institute, La Jolla). Functional enrichment analysis highlighted the involvement of these genes in immune and inflammation-related pathways. To validate these findings, molecular docking analyses were employed to assess the interactions between key compounds and hub targets, using AutoDock Vina 1.1.2 (The Scripps Research Institute, La Jolla) for binding affinity evaluation and visualization of optimal binding conformations. Additionally, Gromacs 2020 (Royal Institute of Technology, Stockholm, Sweden) was utilized for molecular dynamics simulations to verify the stability of compound–target complexes with the highest binding affinities. The expression and relevance of hub targets were further confirmed through mining GEO datasets, while receiver operating characteristic curve analysis assessed their diagnostic significance in PSO. The study identified 189 active ingredients and 227 targets for ZDYXC, with 148 intersection genes being primarily associated with immune regulation and inflammatory processes. Molecular docking revealed strong affinities between core ZDYXC ingredients, such as vestitol, formononetin, isorhamnetin, kaempferol, luteolin, quercetin, and tanshinone IIA, and hub targets including AKT1, STAT3, ESR1, and TP53. Molecular dynamics confirmed the stability of these interactions. Differential expression and receiver operating characteristic analysis further pointed to STAT3 and ESR1 as hub genes in PSO. This study demonstrates that ZDYXC may treat PSO through multiple ingredients, targets, and pathways, providing a valuable foundation for further mechanistic studies and clinical applications.

## 1. Introduction

Psoriasis (PSO) is a chronic papulosquamous skin disease caused by genetic and environmental factors. It is characterized by red or brownish papules or plaques covered with silvery-white scales, often accompanied by itching and pain. Globally, PSO affects approximately 3% of the population, with higher prevalence among Caucasians compared to East Asians and Africans.^[1]^ In China, the prevalence increased from 0.123% in 1984 to 0.47% in 2008.^[2]^ Current treatments include retinoids, immunosuppressants, corticosteroids, and biologics, but these have limitations such as local application constraints, drug dependence, numerous contraindications, and high costs.^[3]^

Traditional Chinese medicine offers multi-component and multi-target treatments with minimal side effects and long-term stability.^[4]^ Zidan Yinxie capsules (ZDYXC), a Chinese patent medicine, consist of Jiangxiang, Danshen, Fuzi, Juemingzi and other herbs, which have complementary effects such as promoting blood circulation, moistening dryness, relieving itching, dispelling wind, and nourishing blood.^[5]^ Previous studies have shown that ZDYXC can effectively alleviate skin itching, reduce the severity of skin lesions, and improve the quality of life in PSO patients.^[5,6]^ However, the specific mechanisms remain unclear.

This study aimed to identify the active components of ZDYXC and PSO-related targets using a network pharmacology approach, and to evaluate the binding affinities between hub targets and compounds through molecular docking. Furthermore, molecular dynamics simulations were performed to estimate the binding stability and interaction strength between the compounds and proteins. The expression and disease-predictive value of hub targets were validated using GEO datasets. These findings may provide insights for future experimental research and clinical applications.

## 2. Materials and methods

**Figure 1.**
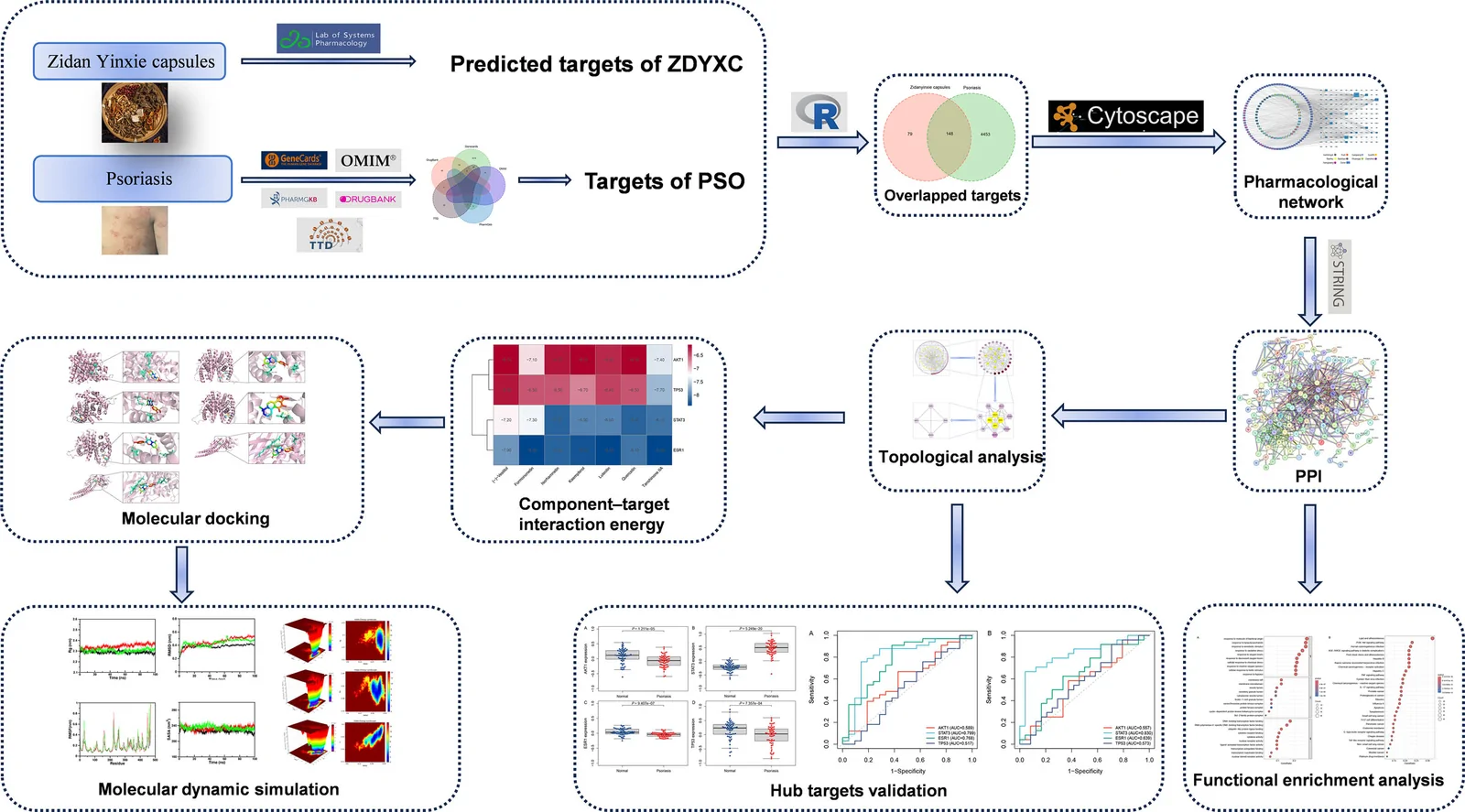
Flowchart of this study. OMIM = Online Mendelian Inheritance in Man, PPI = protein–protein interaction, PSO = psoriasis.

A flowchart of the research process is shown in Figure 1.

### 2.1. Screening for ZDYXC compounds and targets

The TCMSP database (https://tcmsp-e.com/) was used to identify the active ingredients and potential targets of ZDYXC. The search included 9 traditional Chinese medicines: Juemingzi, Fuzi, Ganjiang, Guizhi, Baizhu, Baishao, Huangqi, Danshen, and Jiangxiang. Active ingredients were filtered based on oral bioavailability ≥ 30% and drug-likeness ≥ 0.18. Potential targets were standardized into official symbols using UniProt (http://www.uniprot.org/).

### 2.2. Screening for PSO-related targets

PSO-related targets were identified by searching “Psoriasis” in GeneCards (https://www.genecards.org/), Online Mendelian Inheritance in Man (https://www.omim.org), Pharmacogenetics and Pharmacogenomics Knowledge Base (https://www.pharmgkb.org/), Therapeutic Target Database (https://db.idrblab.net/ttd/), and Drugbank database (https://go.drugbank.com/). The species was set to *Homo sapiens*.

### 2.3. Construction of the ingredients-targets network

The intersection genes of PSO targets and ZDYXC targets were analyzed using the Venn package. An interaction network of active ingredients and target genes was created with Cytoscape (3.9.1), where the size of each target node reflects the number of related events. Drug nodes are differentiated by colors representing various medicine ingredients.

### 2.4. Construction of protein–protein interaction (PPI) network and screening of hub proteins

A PPI network was constructed using the String database (https://cn.string-db.org/). The CytoNCA plugin in Cytoscape was used for topological analysis to identify hub targets based on 6 established indicators. Higher values for each indicator signify greater importance of the node in the network, allowing for the identification of hub networks.

### 2.5. Functional enrichment analysis

Functional enrichment analysis on intersection genes was conducted using the “clusterProfiler” package, including gene ontology (GO) and Kyoto Encyclopedia of Genes and Genomes (KEGG) analyses (with a differential threshold of *P* < .05). Results were visualized using the “ggplot2” package.

### 2.6. Molecular docking

Molecular docking was performed between the hub targets and the primary active compounds, chosen for their degree being more than twice the average value. The 2D structures of these compounds were downloaded from the PubChem database and converted into 3D models using ChemBio3D ultra 14.0 (PerkinElmer Inc., Waltham), followed by optimization for minimal free energy. Protein structures were retrieved from the Protein Data Bank database, with water molecules and small ligands removed using PyMOL 4.6.0 (Schrödinger LLC, New York). The receptor structures were converted to pdbqt format with AutoDockTools. Docking simulations were conducted using AutoDock Vina (1.1.2) to evaluate ligand–receptor binding positions and energies. We visualized the binding position with the lowest energy using PyMOL and created a heatmap of the docking results with R 4.3.2 software (R Foundation for Statistical Computing, Vienna, Austria).

### 2.7. Molecular dynamics simulation

To assess the binding capability and stability of protein–ligand complexes, the top 3 complexes ranked by absolute binding energy were subjected to 100 ns molecular dynamics simulations using GROMACS 2020. The Amber99sb-ildn force field was applied to the proteins, and the generalized AMBER force field 2 force field was used for the ligands. The systems were solvated using the simple point charge extended model water model in a dodecahedral water box containing transferable intermolecular potential 3 point water molecules, and sodium and chloride ions were added to maintain charge neutrality. Prior to the production molecular dynamics simulations, energy minimization was performed for 50,000 steps using the conjugate gradient algorithm. This was followed by equilibration with 100 ps of constant volume and temperature (NVT) at 300 K and 100 ps of constant pressure and temperature (NPT) at 1 bar. The final production simulations were carried out for 100 ns under ambient temperature and pressure. Throughout the simulation, several metrics were analyzed, including root mean square deviation (RMSD), root mean square fluctuation (RMSF), radius of gyration (Rg), the number of hydrogen bonds (H-bonds) between the protein and ligand in each complex, solvent-accessible surface area (SASA), and the Gibbs free energy landscape (FEL). Specifically, RMSD was used to evaluate the stability of the protein–ligand complexes, with lower RMSD values indicating smaller overall structural changes and greater stability. RMSF reflected fluctuations of individual amino acid residues during the simulation, where higher values indicated greater mobility and lower values suggested more restricted movement. The Rg was used to characterize the compactness and stability of the structure, with larger Rg values implying an expanded conformation while smaller values indicated a tightly packed and stable structure. SASA was used to assess the folding and stability of the protein, and a stable SASA curve indicated consistent structural integrity throughout the simulation.

### 2.8. GEO dataset validation and receiver operating characteristic (ROC) curve evaluation

The GSE13355 dataset from the GEO database was used to validate the expression levels of hub targets in 58 PSO patients and 64 healthy controls. To further evaluate the discrimination ability of these targets in PSO, we used GSE14905, which includes 21 normal healthy controls and 33 PSO patients, and GSE41662, consisting of 24 paired samples from lesional and non-lesional skin of PSO patients, as external validation datasets. ROC curves were plotted, and the area under the curve (AUC) was calculated.

## 3. Results

### 3.1. Screening of active ingredients and targets for ZDYXC

A total of 189 active ingredients were screened for ZDYXC, including 14 from Juemingzi, 21 from Fuzi, 5 from Ganjiang, 7 from Guizhi, 7 from Baizhu, 13 from Baishao, 20 from Huangqi, 65 from Danshen, and 37 from Jiangxiang (Table S1, Supplemental Digital Content, https://links.lww.com/MD/P979). We obtained 2437 targets for ZDYXC, after removing duplicates and ineffective targets, resulting in a final count of 227.

### 3.2. PSO genes screening

**Figure 2.**
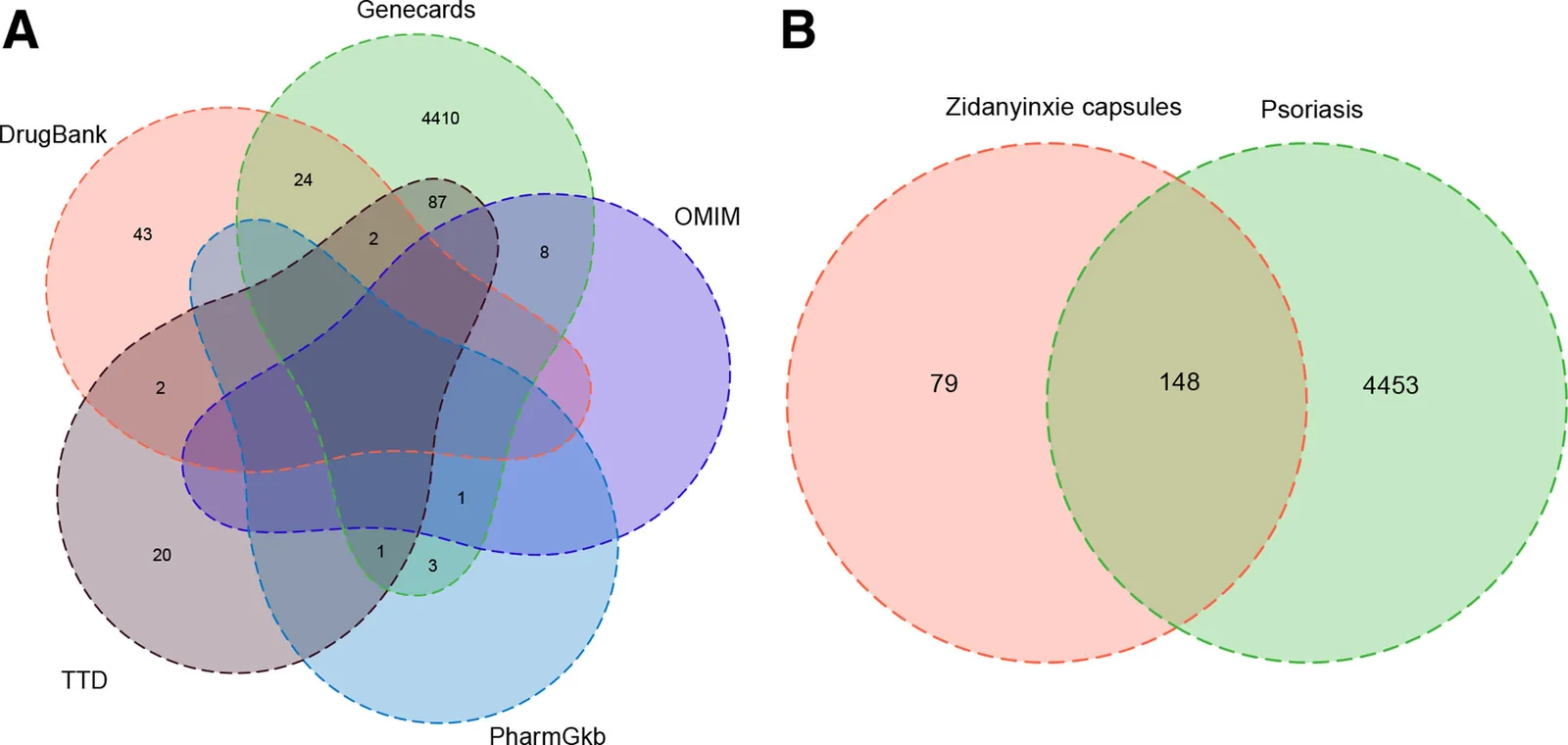
Gene screening. (A) Psoriasis related genes. (B) Zidan Yinxie capsules-psoriasis intersection genes. OMIM = Online Mendelian Inheritance in Man, PharmGkb = Pharmacogenetics and Pharmacogenomics Knowledge Base, TTD = Therapeutic Target Database.

We retrieved 4749 PSO-related genes from 5 databases (GeneCards, 4536; OMIM, 13; PharmGkb, 5; TTD, 112; and DrugBank, 83). After removing duplicate genes, we identified 4601 targets and generated a Venn diagram (Fig. 2A). After matching the targets of ZDYXC active ingredients with PSO-related genes, we obtained 148 intersecting genes (Fig. 2B).

### 3.3. ZDYXC-PSO network construction

**Figure 3.**
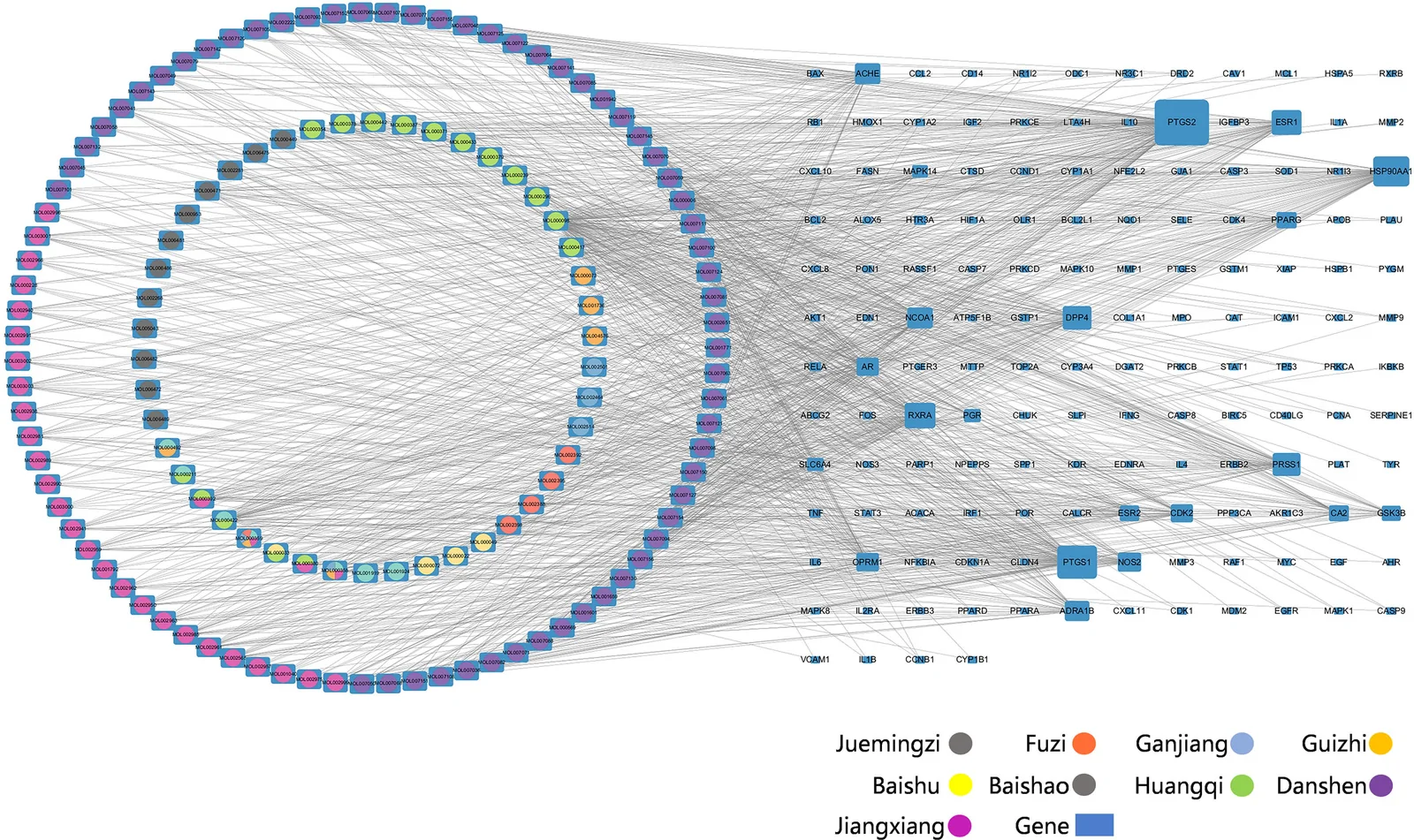
Zidan Yinxie capsules-psoriasis network. Circles indicates active ingredients of Zidan Yinxie capsules (different colors indicates different ingredients), and squares indicates genes. The larger the target, the stronger the association with the ingredients of Zidan Yinxie capsules.

A network comprising 189 active ingredients and 148 intersection genes was constructed using Cytoscape (Fig. 3). In this network, node size reflects the number of connected active ingredients, indicating the target’s importance (degree value). Danshen showed the greatest overlap with disease targets, with quercetin linked to the most targets (Table S2, Supplemental Digital Content, https://links.lww.com/MD/P979).

### 3.4. PPI network construction and hub gene screening

**Figure 4.**
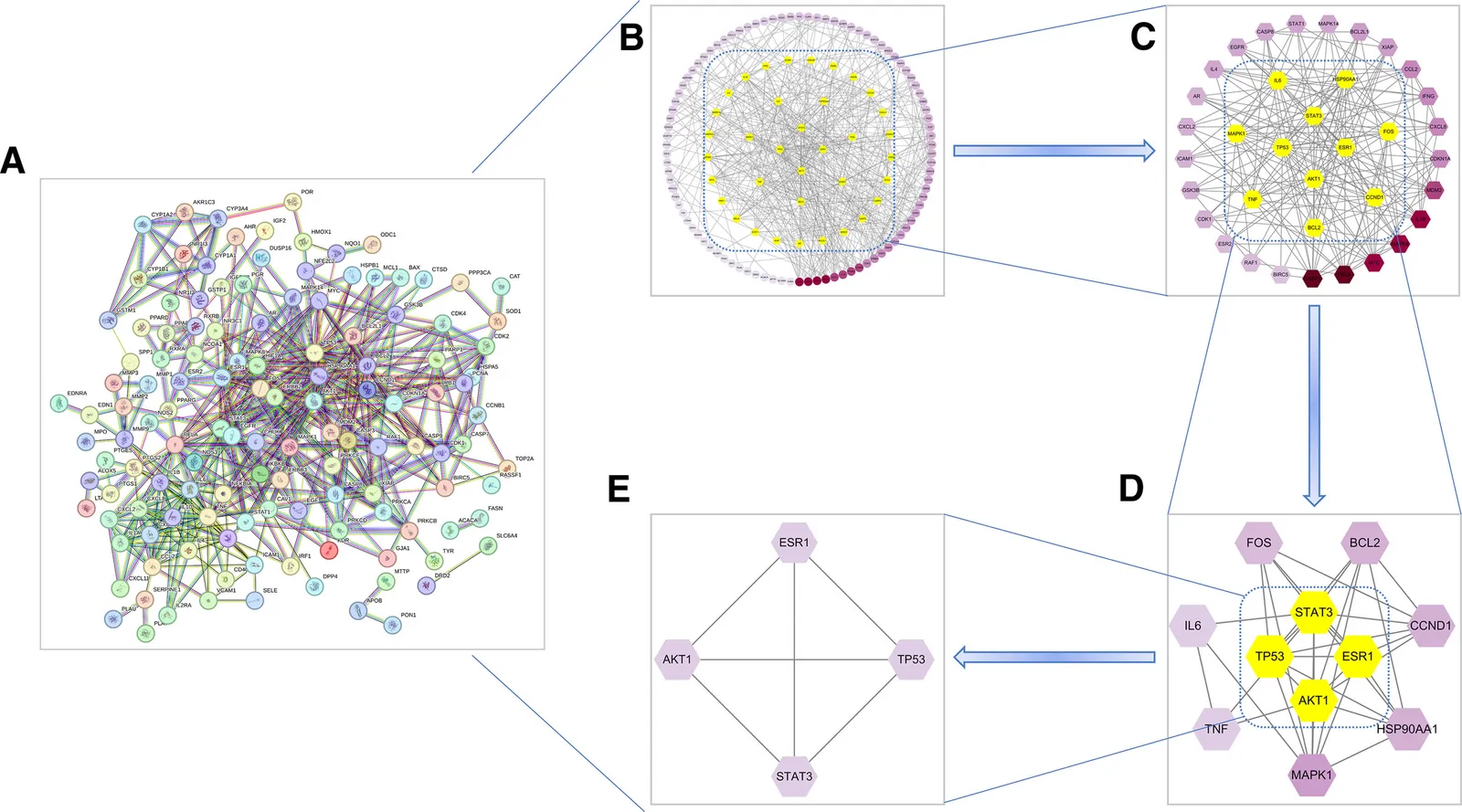
Protein–protein interaction (PPI) network of Zidan Yinxie capsules-psoriasis intersection genes. (A) PPI network of intersection genes. (B–E) Hub gene screening using CytoNCA plug-in.

A PPI network was constructed for the 148 intersecting genes by STRING online analysis tool, which consists of 130 nodes and 493 edges (Fig. 4A). The topological analysis identified 4 hub targets: AKT1, STAT3, ESR1, and TP53 (Fig. 4B–E). The topological scores of these hub genes are presented in Table S3, Supplemental Digital Content, https://links.lww.com/MD/P979.

### 3.5. Functional enrichment analysis

**Figure 5.**
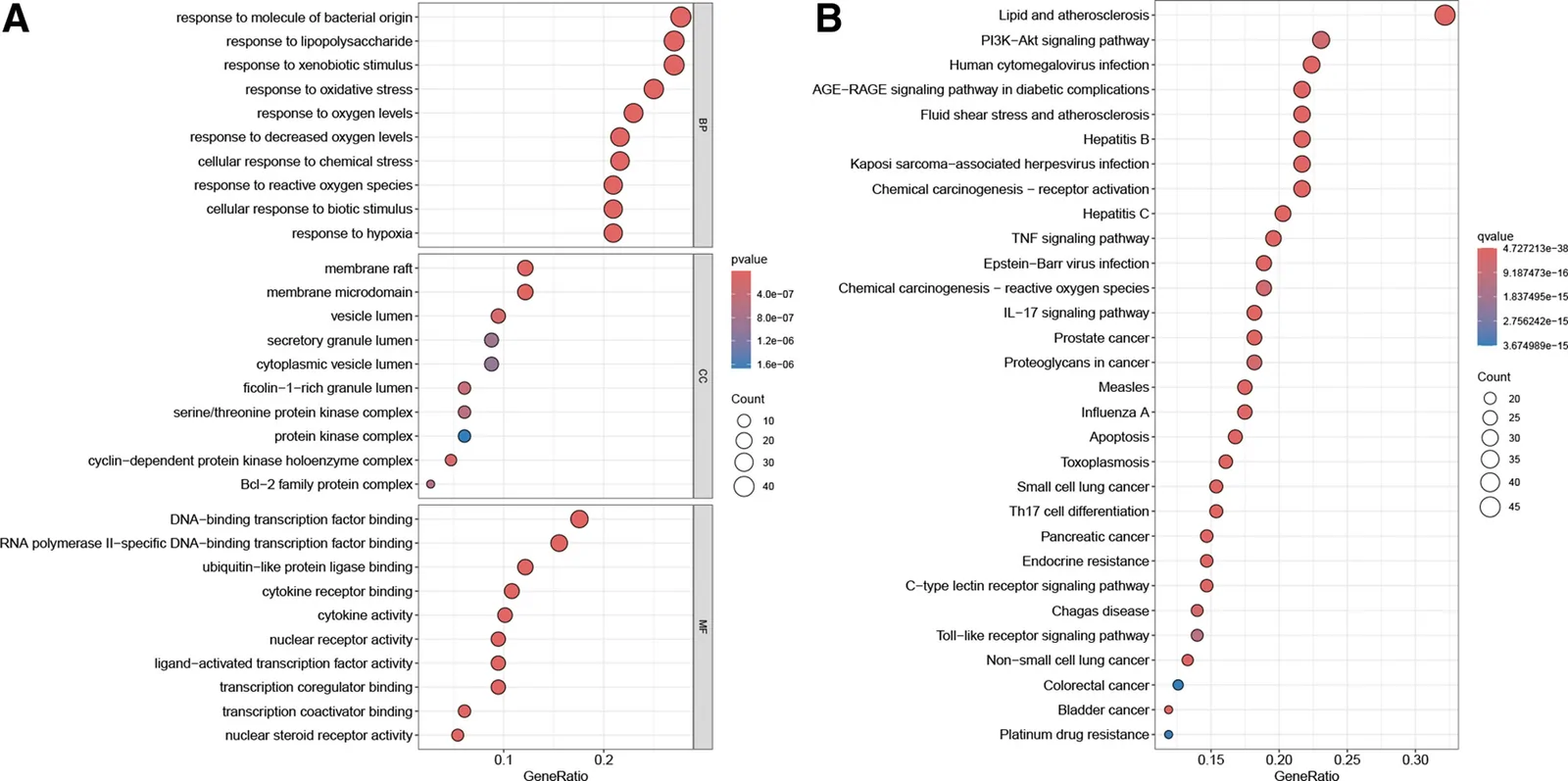
Functional enrichment analysis. (A) GO enrichment analysis results (top 10 of biological processes, cellular components, and molecular functions). (B) KEGG pathway analysis findings (top 20). GO = gene ontology, KEGG = Kyoto Encyclopedia of Genes and Genomes.

GO analysis of the intersection genes yielded a total of 2679 items, comprising 2409 items for biological processes (BP), 81 for cellular components, and 189 for molecular functions. The top 10 mainly enriched items of GO were visualized, respectively (Fig. 5A). In BP, targets were mainly enriched in response to oxidative stress, oxygen levels and hypoxia. Regarding cellular components, the targets were mainly distributed in membrane rafts, microdomains, and protein kinase complexes. In molecular functions, the targets were associated with cytokine activity, nuclear receptor activity and cytokine receptor binding.

KEGG analysis identified 168 signaling pathways related to inflammation and immune response, including the IL-17 signaling pathway and TNF signaling pathway. Figure 5B illustrates the top 20 enriched pathways.

### 3.6. Molecular docking

**Figure 6.**
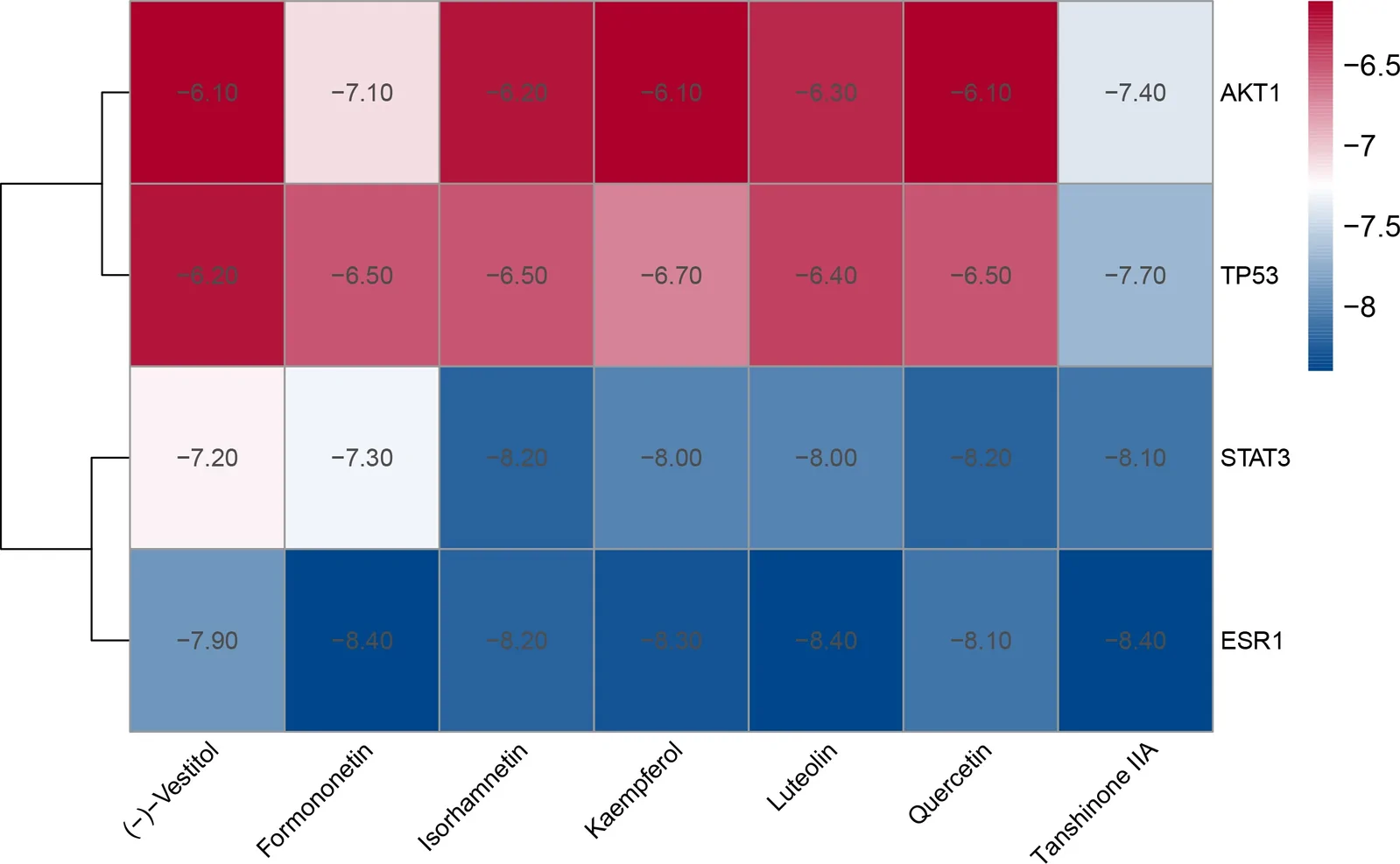
Heatmap of the molecular docking between the active ingredients of Zidan Yinxie capsules and hub targets.

**Figure 7.**
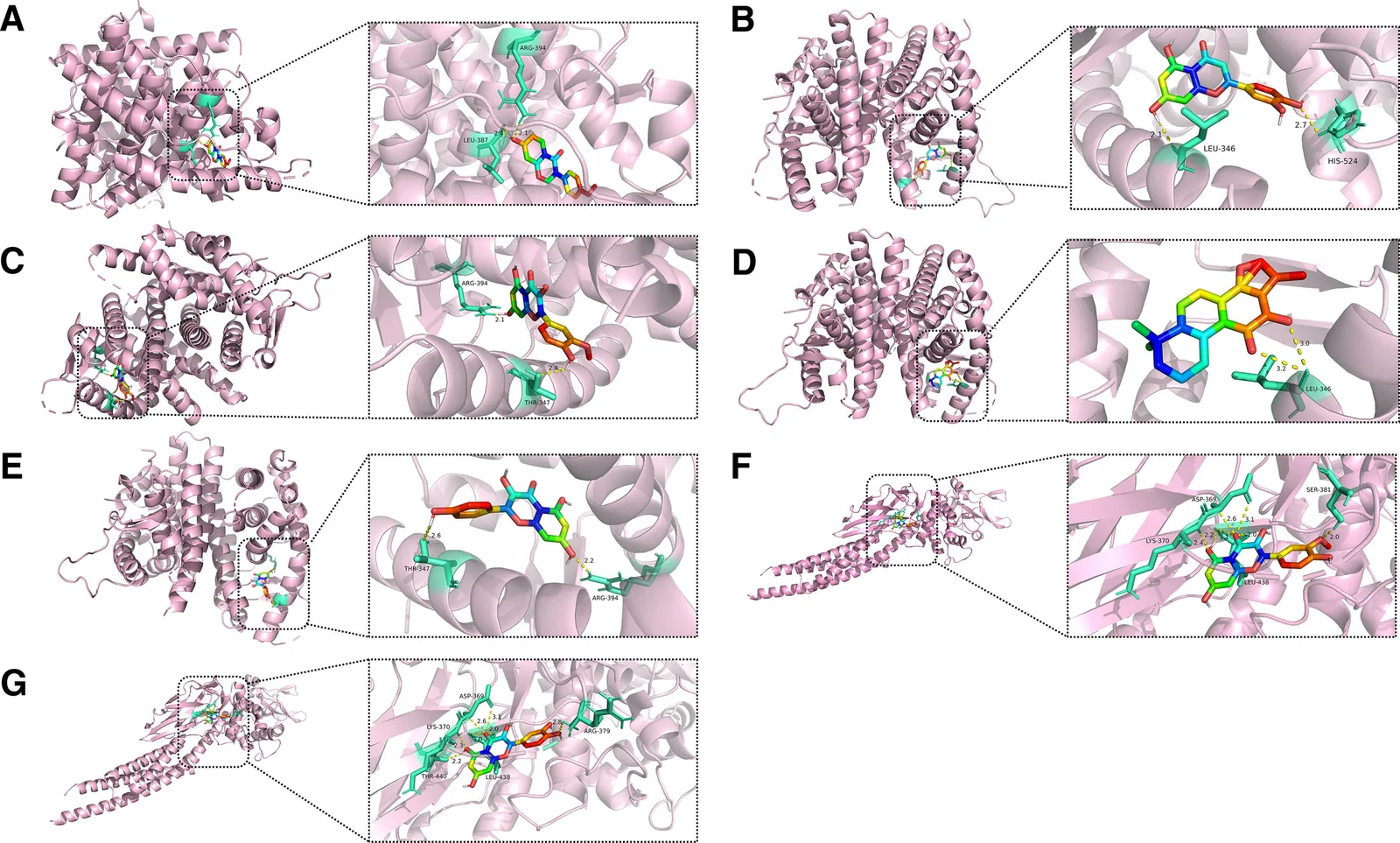
The docking model of the active ingredients and hub targets. (A) Formononetin and ESR1; (B) luteolin and ESR1; (C) isorhamnetin and ESR1; (D) tanshinone IIA and ESR1; (E) kaempferol and ESR1; (F) isorhamnetin and STAT3; and (G) quercetin and STAT3.

The 7 active ingredients were individually docked with 4 hub targets (i.e. AKT1, STAT3, ESR1, and TP53). The binding energies ranged from −6.1 to −9.2 kcal/mol (Fig. 6), suggesting a strong binding affinity and high compatibility between the ingredients and targets.^[7]^ The 7 molecular docking conformations demonstrating the lowest binding energy was chosen and visualized (Fig. 7).

### 3.7. Molecular dynamics

**Figure 8.**
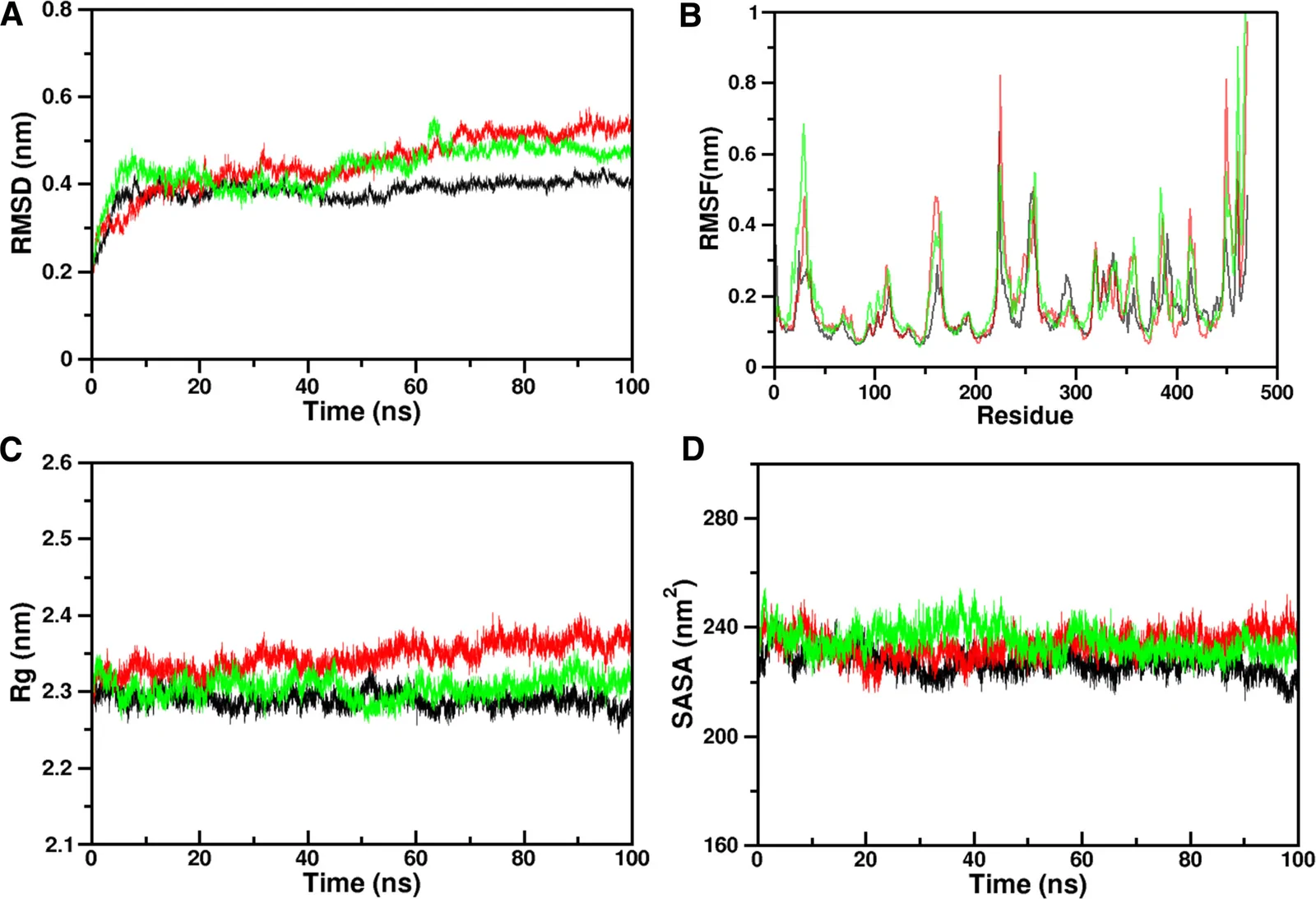
Analysis results of molecular dynamics simulations. (A) RMSD analysis; (B) RMSF analysis; (C) Rg analysis; and (D) SASA analysis. Rg = radius of gyration, RMSD = root mean square deviation, RMSF = root mean square fluctuation, SASA = solvent-accessible surface area.

The 3 molecular complexes exhibiting the strongest binding energies were ESR1 protein complexes with formononetin, tanshinone IIA, and luteolin (−8.4 kcal/mol). To further investigate the interaction strength and stability beyond the limitations of semi-flexible molecular docking (which cannot account for protein flexibility), we performed 100 ns molecular dynamics simulations on these ESR1 complexes. As shown in Figure 8A, all 3 complexes demonstrated excellent stability with RMSD fluctuations below 1 nm. Figure 8B revealed nearly overlapping RMSF profiles across all groups within a 1 nm range, indicating minimal impact on amino acid residue stability comparable to the co-crystallized ligand. Figure 8C displayed stable Rg trajectories (2.3 nm fluctuation range) with overlapping curves, confirming maintained structural compactness. The SASA analysis in Figure 8D showed consistent profiles with 240 nm² fluctuations, demonstrating preserved structural integrity throughout simulations. These comprehensive analyses confirm that ESR1 forms tightly packed, stable complexes with formononetin, tanshinone IIA, and luteolin without significant alterations to protein conformation, exhibiting stability parameters equivalent to the co-crystallized ligand control.

**Figure 9.**
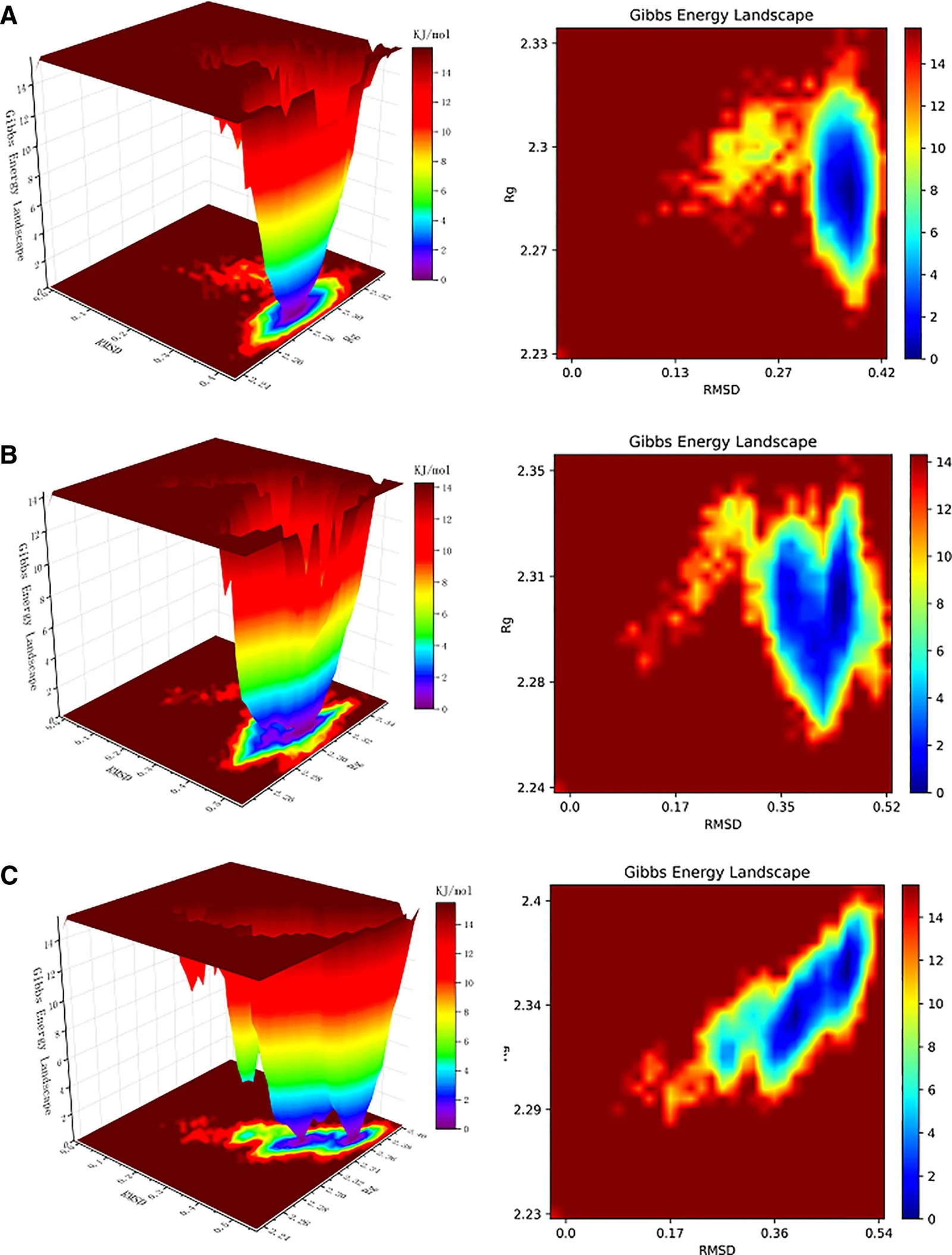
Gibbs free energy landscape map of the complex. (A) Formononetin and ESR1; (B) tanshinone IIA and ESR1; and (C) luteolin and ESR1.

Figure 9 demonstrates that the minimum-energy clusters in the FEL exhibit optimal single and smooth degree, indicating robust structural stability of the ESR1 complexes formed with formononetin, tanshinone IIA, and luteolin.

### 3.8. Hub targets validation

**Figure 10.**
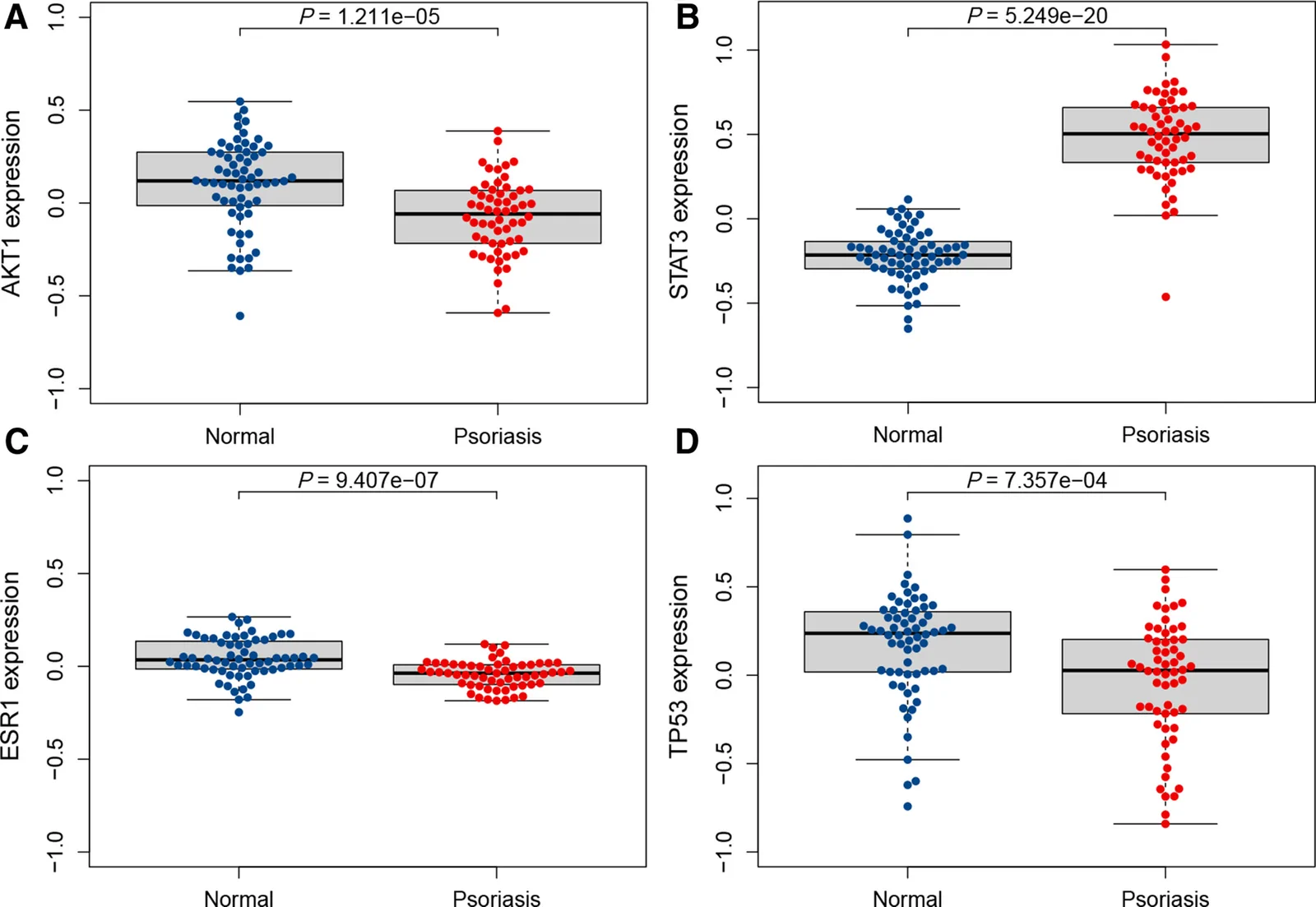
Expression levels of hub targets in the GSE13355 dataset. (A) AKT1 expression; (B) STAT3 expression; (C) ESR1 expression; and (D) TP53 expression.

**Figure 11.**
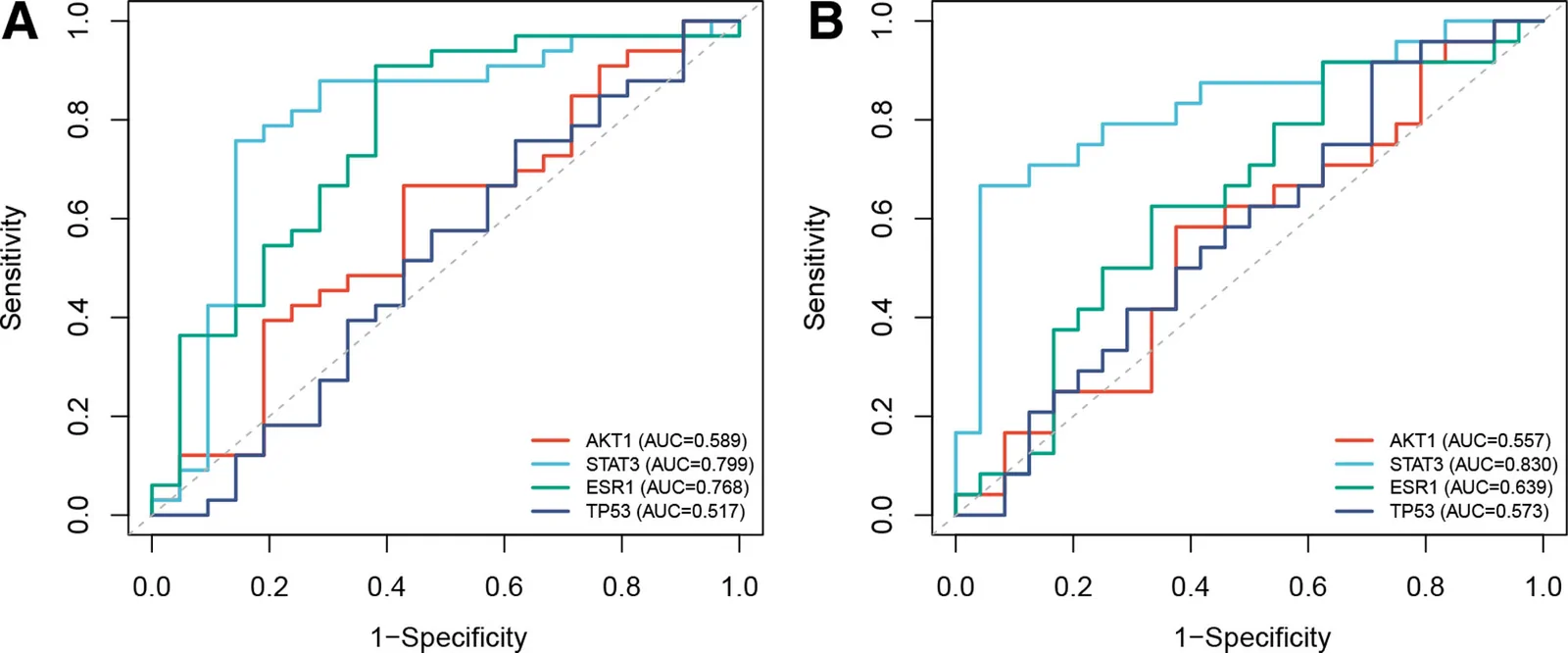
ROC curves of AKT1, STAT3, ESR1, and TP53 in the GSE14905 (A) and GSE41662 datasets (B). ROC = receiver operating characteristic.

Expression analysis of hub targets in the GSE13355 dataset showed significant differences between normal and PSO groups (Fig. 10). ROC curves were generated for these targets, and the AUC values indicated that STAT3 and ESR1 exhibited high AUC values in both PSO versus healthy controls and PSO lesions versus non-lesions within the GEO datasets, suggesting their crucial roles in PSO (Fig. 11).

## 4. Discussion

This study investigated the potential mechanisms of ZDYXC in treating PSO using network pharmacology and molecular docking. Key compounds (quercetin, luteolin, and tanshinone IIA) were identified to interact with hub targets (e.g., STAT3, ESR1). Functional enrichment and docking analysis, along with external validation, supported their relevance in PSO pathophysiology.

Quercetin, a natural flavonoid compound, has the potential to modulate various intracellular and extracellular signaling pathways implicated in disease progression. It is regarded as a promising natural agent for disease prevention and treatment.^[8]^ Previous studies have suggested that the anti-psoriatic mechanism of quercetin may be associated with its anti-inflammatory and antioxidant properties, downregulation of miR-155, which is highly expressed in PSO, and inhibition of the NF-κB signaling pathway.^[9–11]^ Luteolin, another natural flavonoid, has been extensively researched and shown to possess various pharmacological effects, including anti-inflammatory, antioxidant, immunomodulatory, and anticancer properties.^[12–14]^ Studies by Kim et al have reported that luteolin and similar compounds can reduce the proliferation of keratinocytes.^[15,16]^ Additionally, research by Zhou et al suggests that luteolin can alleviate PSO-like lesions induced by imiquimod in mice, possibly by influencing the phosphorylation and expression of NF-κB, downregulating relevant mediators and inflammatory factors in the disease process.^[17]^ Kaempferol has been shown to possess anti-inflammatory, antidiabetic, and anticancer properties.^[18]^ Research also indicates that kaempferol can alleviate PSO-like skin lesions in mice induced by imiquimod via reducing the expression of key pro-inflammatory cytokine genes, infiltration of CD3 + T cells, inhibiting the development of Th17 cells, and suppressing the skin pro-inflammatory NF-κB signaling pathway.^[19]^ Tanshinone IIA, a diterpenoid compound, exerts anti-inflammatory and immunomodulatory effects by modulating the TLR4/TAK1/NF-κB signaling pathway.^[20,21]^ Furthermore, tanshinone IIA exhibits antitumor properties by inducing cell differentiation and apoptosis.^[22]^ Formononetin has been found to possess various biological functions, such as oxidative stress-reducing, anti-inflammatory, neuroprotective, and cancer-inhibiting.^[23–25]^ Franchin et al found that vestitol inhibits neutrophil migration during the inflammatory process, exerting anti-inflammatory effects.^[26]^ Bueno-Silva et al reached consistent conclusions and additionally found that vestitol reduces the activation of the NF-κB and Erk 1/2 pathways, induces macrophages to enter M2-like polarization, and similarly exhibits anti-inflammatory effects.^[27]^ Isorhamnetin exhibits antioxidant, cardiovascular and cerebrovascular protective, and anticancer effects,^[28–30]^ and can ameliorate PSO-like skin inflammation in mice by reducing levels of inflammatory factors, including TNF-α, IL-17A, and NF-κB in the skin.^[31]^

This study identified 148 targets of ZDYXC in PSO. By constructing a PPI network and further analyzing it, we identified 4 hub targets: AKT1, STAT3, ESR1, and TP53. AKT1 is a serine/threonine protein kinase and one of the AKT subtypes. Research has shown that activation of AKT leads to excessive proliferation of psoriatic keratinocytes.^[32]^ STAT3 is a transcription factor that is essential for regulating numerous BP, such as cell migration, proliferation and differentiation.^[33]^ For instance, studies by Xie et al have shown that overexpression of STAT3 leads to excessive proliferation of keratinocytes, resulting in PSO.^[34]^ ESR1 has been identified as playing a pivotal role in both the initiation and recurrence of PSO, influencing the progression through its involvement in key cellular and molecular processes.^[35]^ The TP53 protein can mediate cell apoptosis through the Bcl-2/Bax pathway, playing an important role in the apoptosis process in psoriatic skin.^[36,37]^

Consistent with previous studies, our research revealed through KEGG pathway analysis that ZDYXC has a broad impact on signaling pathways directly linked with the pathophysiology of PSO, including the TNF signaling pathway, and Th17 cell differentiation.^[38–40]^ Activation of TNF pathway triggers diverse inflammatory responses and immune cell infiltration, promoting aberrant proliferation of keratinocytes and cytokine production, consequently contributing to the formation and exacerbation of psoriatic lesions.^[41]^ The IL-17 signaling pathway is central to the pathogenesis of PSO. Its activation leads to the infiltration of inflammatory and immune cells, promoting aberrant proliferation of keratinocytes and cytokine production.^[42]^ Th17 cells, a specific subset of helper T cells, mediate and sustain inflammatory responses by secreting IL-17 and other pro-inflammatory cytokines.^[43]^ The differentiation and activation of Th17 cells enhance the immune system’s assault on the skin, thereby resulting in the formation and exacerbation of psoriatic lesions.^[44]^

This study identified the main active ingredients of ZDYXC as quercetin, luteolin, kaempferol, tanshinone, formononetin, vestitol, and isorhamnetin, which targeting AKT1, STAT3, ESR1, and TP53. The binding energies between these active compounds and hub genes ranged from −6.10 to −8.40 kcal/mol, all falling below −5 kcal/mol, indicating favorable binding affinity and high compatibility between the compounds and targets. Generally, binding energies under −4.25 kcal/mol suggest a certain level of binding activity, while energies below −5.0 kcal/mol indicate a strong binding affinity, and values below −7.0 kcal/mol reflect a very high binding strength.^[7]^ Molecular dynamics simulation analyses of RMSD, RMSF, Rg, SASA, and FEL demonstrated that the complexes formed by formononetin, tanshinone IIA, and luteolin with ESR1 exhibited notable stability and high binding strength, and were capable of forming relatively stable hydrogen bond interactions. These findings suggest that formononetin, tanshinone IIA, and luteolin serve as the main active constituents through which ZDYXC exerts its therapeutic action against PSO, principally via ESR1 as a key molecular target.

Using GEO validation datasets to evaluate the expression levels and discrimination ability of hub targets, we found significant differences in the expression of AKT1, STAT3, ESR1, and TP53 between normal and disease groups. STAT3 and ESR1 demonstrated higher AUC values, indicating that these hub targets are potential candidates for the diagnosis and treatment of PSO. Further validation of their expression and related functions through mutation analysis and RT-PCR is warranted.

Network pharmacology integrates computational, experimental, and clinical approaches to understand the complex biological basis of diseases and traditional chinese medicine treatments.^[45]^ Our study used network pharmacology to investigate potential targets and specific mechanisms of ZDYXC in the treatment of PSO. However, there are several limitations. Firstly, while all data were sourced from public databases and analyzed through comprehensive bioinformatics methods, variations in database management could introduce selection bias. Secondly, due to the complexity of ingredients, proteins, and pathways associated with Chinese medicine, more advanced algorithmic support is necessary for a thorough understanding of its mechanisms. Lastly, although our study does not include experimental validation, it offers a potential pathway for future in vitro and in vivo research to investigate the molecular mechanisms by which ZDYXC may treat PSO.

## 5. Conclusion

In this study, we utilized network pharmacology to investigate the active ingredients, hub targets, and key pathways associated with the treatment of PSO by ZDYXC, and further validated our findings through molecular docking, molecular dynamics and external datasets. The results indicate that ZDYXC exerts its therapeutic effects on PSO through multiple components, targets, and signaling pathways, thereby laying a foundation for understanding its mechanisms of action and guiding future experimental research and clinical applications.

## Author contributions

**Conceptualization:** Jianhua Bu, Xianwei Wu.

**Data curation:** Caiqing Mao.

**Formal analysis:** Jianhua Bu, Lisheng Nan.

**Funding acquisition:** Xianwei Wu.

**Investigation:** Caiqing Mao, Lisheng Nan.

**Methodology:** Jianhua Bu.

**Software:** Jianhua Bu, Caiqing Mao.

**Supervision:** Xianwei Wu.

**Validation:** Lisheng Nan.

**Visualization:** Xianwei Wu.

**Writing – original draft:** Jianhua Bu.

**Writing – review & editing:** Xianwei Wu.

## Supplementary Material
