# Supplementary material for: Integrative analysis of Zidan Yinxie capsules for psoriasis treatment: Insights from network pharmacology, molecular dynamics simulation, and GEO database analysis
Source: Medicine (Baltimore). 2025 Sep 26;104(39):e44446. doi: 10.1097/MD.0000000000044446 (PMC13593010; doi:10.1097/MD.0000000000044446)
Supplement: Supplementary file 1 [file medi-104-e44446-s001.docx]

**Table S1.** Details of 189 efficacious active ingredients in Zidan Psoriasis Capsules.

| Drug | Mol ID | Molecule Name | OB(%) | DL |
| --- | --- | --- | --- | --- |
| Baishao | MOL001910 | 11alpha,12alpha-epoxy-3beta-23-dihydroxy-30-norolean-20-en-28,12beta-olide | 64.77 | 0.38 |
|  | MOL001918 | paeoniflorgenone | 87.59 | 0.37 |
|  | MOL001919 | (3S,5R,8R,9R,10S,14S)-3,17-dihydroxy-4,4,8,10,14-pentamethyl-2,3,5,6,7,9-hexahydro-1H-cyclopenta[a]phenanthrene-15,16-dione | 43.56 | 0.53 |
|  | MOL001921 | Lactiflorin | 49.12 | 0.8 |
|  | MOL001924 | paeoniflorin | 53.87 | 0.79 |
|  | MOL001925 | paeoniflorin_qt | 68.18 | 0.4 |
|  | MOL001928 | albiflorin_qt | 66.64 | 0.33 |
|  | MOL001930 | benzoyl paeoniflorin | 31.27 | 0.75 |
|  | MOL000211 | Mairin | 55.38 | 0.78 |
|  | MOL000358 | beta-sitosterol | 36.91 | 0.75 |
|  | MOL000359 | sitosterol | 36.91 | 0.75 |
|  | MOL000422 | kaempferol | 41.88 | 0.24 |
|  | MOL000492 | (+)-catechin | 54.83 | 0.24 |
| Baizhu | MOL000020 | 12-senecioyl-2E,8E,10E-atractylentriol | 62.4 | 0.22 |
|  | MOL000021 | 14-acetyl-12-senecioyl-2E,8E,10E-atractylentriol | 60.31 | 0.31 |
|  | MOL000022 | 14-acetyl-12-senecioyl-2E,8Z,10E-atractylentriol | 63.37 | 0.3 |
|  | MOL000028 | α-Amyrin | 39.51 | 0.76 |
|  | MOL000033 | (3S,8S,9S,10R,13R,14S,17R)-10,13-dimethyl-17-[(2R,5S)-5-propan-2-yloctan-2-yl]-2,3,4,7,8,9,11,12,14,15,16,17-dodecahydro-1H-cyclopenta[a]phenanthren-3-ol | 36.23 | 0.78 |
|  | MOL000049 | 3β-acetoxyatractylone | 54.07 | 0.22 |
|  | MOL000072 | 8β-ethoxy atractylenolide Ⅲ | 35.95 | 0.21 |
| Danshen | MOL001601 | 1,2,5,6-tetrahydrotanshinone | 38.75 | 0.36 |
|  | MOL001659 | Poriferasterol | 43.83 | 0.76 |
|  | MOL001771 | poriferast-5-en-3beta-ol | 36.91 | 0.75 |
|  | MOL001942 | isoimperatorin | 45.46 | 0.23 |
|  | MOL002222 | sugiol | 36.11 | 0.28 |
|  | MOL002651 | Dehydrotanshinone II A | 43.76 | 0.4 |
|  | MOL002776 | Baicalin | 40.12 | 0.75 |
|  | MOL000569 | digallate | 61.85 | 0.26 |
|  | MOL000006 | luteolin | 36.16 | 0.25 |
|  | MOL006824 | α-amyrin | 39.51 | 0.76 |
|  | MOL007036 | 5,6-dihydroxy-7-isopropyl-1,1-dimethyl-2,3-dihydrophenanthren-4-one | 33.77 | 0.29 |
|  | MOL007041 | 2-isopropyl-8-methylphenanthrene-3,4-dione | 40.86 | 0.23 |
|  | MOL007045 | 3α-hydroxytanshinoneⅡa | 44.93 | 0.44 |
|  | MOL007048 | (E)-3-[2-(3,4-dihydroxyphenyl)-7-hydroxy-benzofuran-4-yl]acrylic acid | 48.24 | 0.31 |
|  | MOL007049 | 4-methylenemiltirone | 34.35 | 0.23 |
|  | MOL007050 | 2-(4-hydroxy-3-methoxyphenyl)-5-(3-hydroxypropyl)-7-methoxy-3-benzofurancarboxaldehyde | 62.78 | 0.4 |
|  | MOL007051 | 6-o-syringyl-8-o-acetyl shanzhiside methyl ester | 46.69 | 0.71 |
|  | MOL007058 | formyltanshinone | 73.44 | 0.42 |
|  | MOL007059 | 3-beta-Hydroxymethyllenetanshiquinone | 32.16 | 0.41 |
|  | MOL007061 | Methylenetanshinquinone | 37.07 | 0.36 |
|  | MOL007063 | przewalskin a | 37.11 | 0.65 |
|  | MOL007064 | przewalskin b | 110.32 | 0.44 |
|  | MOL007068 | Przewaquinone B | 62.24 | 0.41 |
|  | MOL007069 | przewaquinone c | 55.74 | 0.4 |
|  | MOL007070 | (6S,7R)-6,7-dihydroxy-1,6-dimethyl-8,9-dihydro-7H-naphtho[8,7-g]benzofuran-10,11-dione | 41.31 | 0.45 |
|  | MOL007071 | przewaquinone f | 40.31 | 0.46 |
|  | MOL007077 | sclareol | 43.67 | 0.21 |
|  | MOL007079 | tanshinaldehyde | 52.47 | 0.45 |
|  | MOL007081 | Danshenol B | 57.95 | 0.56 |
|  | MOL007082 | Danshenol A | 56.97 | 0.52 |
|  | MOL007085 | Salvilenone | 30.38 | 0.38 |
|  | MOL007088 | cryptotanshinone | 52.34 | 0.4 |
|  | MOL007093 | dan-shexinkum d | 38.88 | 0.55 |
|  | MOL007094 | danshenspiroketallactone | 50.43 | 0.31 |
|  | MOL007098 | deoxyneocryptotanshinone | 49.4 | 0.29 |
|  | MOL007100 | dihydrotanshinlactone | 38.68 | 0.32 |
|  | MOL007101 | dihydrotanshinoneⅠ | 45.04 | 0.36 |
|  | MOL007105 | epidanshenspiroketallactone | 68.27 | 0.31 |
|  | MOL007107 | C09092 | 36.07 | 0.25 |
|  | MOL007108 | isocryptotanshi-none | 54.98 | 0.39 |
|  | MOL007111 | Isotanshinone II | 49.92 | 0.4 |
|  | MOL007115 | manool | 45.04 | 0.2 |
|  | MOL007118 | microstegiol | 39.61 | 0.28 |
|  | MOL007119 | miltionone Ⅰ | 49.68 | 0.32 |
|  | MOL007120 | miltionone Ⅱ | 71.03 | 0.44 |
|  | MOL007121 | miltipolone | 36.56 | 0.37 |
|  | MOL007122 | Miltirone | 38.76 | 0.25 |
|  | MOL007123 | miltirone Ⅱ | 44.95 | 0.24 |
|  | MOL007124 | neocryptotanshinone ii | 39.46 | 0.23 |
|  | MOL007125 | neocryptotanshinone | 52.49 | 0.32 |
|  | MOL007127 | 1-methyl-8,9-dihydro-7H-naphtho[5,6-g]benzofuran-6,10,11-trione | 34.72 | 0.37 |
|  | MOL007130 | prolithospermic acid | 64.37 | 0.31 |
|  | MOL007132 | (2R)-3-(3,4-dihydroxyphenyl)-2-[(Z)-3-(3,4-dihydroxyphenyl)acryloyl]oxy-propionic acid | 109.38 | 0.35 |
|  | MOL007140 | (Z)-3-[2-[(E)-2-(3,4-dihydroxyphenyl)vinyl]-3,4-dihydroxy-phenyl]acrylic acid | 88.54 | 0.26 |
|  | MOL007141 | salvianolic acid g | 45.56 | 0.61 |
|  | MOL007142 | salvianolic acid j | 43.38 | 0.72 |
|  | MOL007143 | salvilenone Ⅰ | 32.43 | 0.23 |
|  | MOL007145 | salviolone | 31.72 | 0.24 |
|  | MOL007149 | NSC 122421 | 34.49 | 0.28 |
|  | MOL007150 | (6S)-6-hydroxy-1-methyl-6-methylol-8,9-dihydro-7H-naphtho[8,7-g]benzofuran-10,11-quinone | 75.39 | 0.46 |
|  | MOL007151 | Tanshindiol B | 42.67 | 0.45 |
|  | MOL007152 | Przewaquinone E | 42.85 | 0.45 |
|  | MOL007154 | tanshinone iia | 49.89 | 0.4 |
|  | MOL007155 | (6S)-6-(hydroxymethyl)-1,6-dimethyl-8,9-dihydro-7H-naphtho[8,7-g]benzofuran-10,11-dione | 65.26 | 0.45 |
|  | MOL007156 | tanshinone Ⅵ | 45.64 | 0.3 |
| Fuzi | MOL002211 | 11,14-eicosadienoic acid | 39.99 | 0.2 |
|  | MOL002388 | Delphin_qt | 57.76 | 0.28 |
|  | MOL002392 | Deltoin | 46.69 | 0.37 |
|  | MOL002393 | Demethyldelavaine A | 34.52 | 0.18 |
|  | MOL002394 | Demethyldelavaine B | 34.52 | 0.18 |
|  | MOL002395 | Deoxyandrographolide | 56.3 | 0.31 |
|  | MOL002397 | karakoline | 51.73 | 0.73 |
|  | MOL002398 | Karanjin | 69.56 | 0.34 |
|  | MOL002401 | Neokadsuranic acid B | 43.1 | 0.85 |
|  | MOL002406 | 2,7-Dideacetyl-2,7-dibenzoyl-taxayunnanine F | 39.43 | 0.38 |
|  | MOL002410 | benzoylnapelline | 34.06 | 0.53 |
|  | MOL002415 | 6-Demethyldesoline | 51.87 | 0.66 |
|  | MOL002416 | deoxyaconitine | 30.96 | 0.24 |
|  | MOL002419 | (R)-Norcoclaurine | 82.54 | 0.21 |
|  | MOL002421 | ignavine | 84.08 | 0.25 |
|  | MOL002422 | isotalatizidine | 50.82 | 0.73 |
|  | MOL002423 | jesaconitine | 33.41 | 0.19 |
|  | MOL002433 | (3R,8S,9R,10R,13R,14S,17R)-3-hydroxy-4,4,9,13,14-pentamethyl-17-[(E,2R)-6-methyl-7-[(2R,3R,4S,5S,6R)-3,4,5-trihydroxy-6-[[(2R,3R,4S,5S,6R)-3,4,5-trihydroxy-6-(hydroxymethyl)oxan-2-yl]oxymethyl]oxan-2-yl]oxyhept-5-en-2-yl]-1,2,3,7,8,10,12,15,16,17-decahydr | 41.52 | 0.22 |
|  | MOL002434 | Carnosifloside I_qt | 38.16 | 0.8 |
|  | MOL000359 | sitosterol | 36.91 | 0.75 |
|  | MOL000538 | hypaconitine | 31.39 | 0.26 |
| Ganjiang | MOL002464 | 1-Monolinolein | 37.18 | 0.3 |
|  | MOL002501 | [(1S)-3-[(E)-but-2-enyl]-2-methyl-4-oxo-1-cyclopent-2-enyl] (1R,3R)-3-[(E)-3-methoxy-2-methyl-3-oxoprop-1-enyl]-2,2-dimethylcyclopropane-1-carboxylate | 62.52 | 0.31 |
|  | MOL002514 | Sexangularetin | 62.86 | 0.3 |
|  | MOL000358 | beta-sitosterol | 36.91 | 0.75 |
|  | MOL000359 | sitosterol | 36.91 | 0.75 |
| Guizhi | MOL001736 | (-)-taxifolin | 60.51 | 0.27 |
|  | MOL000358 | beta-sitosterol | 36.91 | 0.75 |
|  | MOL000359 | sitosterol | 36.91 | 0.75 |
|  | MOL000492 | (+)-catechin | 54.83 | 0.24 |
|  | MOL000073 | ent-Epicatechin | 48.96 | 0.24 |
|  | MOL004576 | taxifolin | 57.84 | 0.27 |
|  | MOL011169 | Peroxyergosterol | 44.39 | 0.82 |
| Huangqi | MOL000211 | Mairin | 55.38 | 0.78 |
|  | MOL000239 | Jaranol | 50.83 | 0.29 |
|  | MOL000296 | hederagenin | 36.91 | 0.75 |
|  | MOL000033 | (3S,8S,9S,10R,13R,14S,17R)-10,13-dimethyl-17-[(2R,5S)-5-propan-2-yloctan-2-yl]-2,3,4,7,8,9,11,12,14,15,16,17-dodecahydro-1H-cyclopenta[a]phenanthren-3-ol | 36.23 | 0.78 |
|  | MOL000354 | isorhamnetin | 49.6 | 0.31 |
|  | MOL000371 | 3,9-di-O-methylnissolin | 53.74 | 0.48 |
|  | MOL000374 | 5'-hydroxyiso-muronulatol-2',5'-di-O-glucoside | 41.72 | 0.69 |
|  | MOL000378 | 7-O-methylisomucronulatol | 74.69 | 0.3 |
|  | MOL000379 | 9,10-dimethoxypterocarpan-3-O-β-D-glucoside | 36.74 | 0.92 |
|  | MOL000380 | (6aR,11aR)-9,10-dimethoxy-6a,11a-dihydro-6H-benzofurano[3,2-c]chromen-3-ol | 64.26 | 0.42 |
|  | MOL000387 | Bifendate | 31.1 | 0.67 |
|  | MOL000392 | formononetin | 69.67 | 0.21 |
|  | MOL000398 | isoflavanone | 109.99 | 0.3 |
|  | MOL000417 | Calycosin | 47.75 | 0.24 |
|  | MOL000422 | kaempferol | 41.88 | 0.24 |
|  | MOL000433 | FA | 68.96 | 0.71 |
|  | MOL000438 | (3R)-3-(2-hydroxy-3,4-dimethoxyphenyl)chroman-7-ol | 67.67 | 0.26 |
|  | MOL000439 | isomucronulatol-7,2'-di-O-glucosiole | 49.28 | 0.62 |
|  | MOL000442 | 1,7-Dihydroxy-3,9-dimethoxy pterocarpene | 39.05 | 0.48 |
|  | MOL000098 | quercetin | 46.43 | 0.28 |
| Jiangxiang | MOL001040 | (2R)-5,7-dihydroxy-2-(4-hydroxyphenyl)chroman-4-one | 42.36 | 0.21 |
|  | MOL001792 | DFV | 32.76 | 0.18 |
|  | MOL000228 | (2R)-7-hydroxy-5-methoxy-2-phenylchroman-4-one | 55.23 | 0.2 |
|  | MOL002565 | Medicarpin | 49.22 | 0.34 |
|  | MOL002914 | Eriodyctiol (flavanone) | 41.35 | 0.24 |
|  | MOL002938 | (3R)-4'-Methoxy-2',3,7-trihydroxyisoflavanone | 68.86 | 0.27 |
|  | MOL002939 | (3R)-5'-Methoxyvestitol | 83.06 | 0.26 |
|  | MOL002940 | (3R)-3-(2,3-dihydroxy-4-methoxyphenyl)-7-hydroxychroman-4-one | 52.06 | 0.27 |
|  | MOL002941 | (3R)-3-(2,3-dihydroxy-4-methoxyphenyl)chroman-7,8-diol | 82.35 | 0.27 |
|  | MOL002950 | (3R)-7,2',3'-trihydroxy-4-methoxyisoflavan | 69.65 | 0.24 |
|  | MOL002957 | 9-O-Methylcoumestrol | 33.73 | 0.38 |
|  | MOL002958 | 3'-Hydroxymelanettin | 30.69 | 0.27 |
|  | MOL002959 | 3'-Methoxydaidzein | 48.57 | 0.24 |
|  | MOL002961 | (-)-Vestitol | 70.29 | 0.21 |
|  | MOL002962 | (3S)-7-hydroxy-3-(2,3,4-trimethoxyphenyl)chroman-4-one | 48.23 | 0.33 |
|  | MOL002963 | 4',5',7-trimethyl-3-methoxyflavone | 40.66 | 0.25 |
|  | MOL002966 | Dalbergin | 78.18 | 0.2 |
|  | MOL002967 | 7-hydroxy-4'-methoxy-2',5'-dioxo-4-[(3R)-2',7-dihydroxy-4'-methoxyisoflavan-5'-yl]isoflavane | 34.78 | 0.7 |
|  | MOL002973 | Bowdichione | 55.78 | 0.28 |
|  | MOL002975 | butin | 69.94 | 0.21 |
|  | MOL002981 | Duartin | 70.63 | 0.34 |
|  | MOL002982 | (3R,4R)-3',7-dihydroxy-2',4'-dimethoxy-4-[(2S)-4',5,7-trihydroxyflavanone-6-yl]isoflavan | 33.96 | 0.63 |
|  | MOL002985 | isoduartin | 74.11 | 0.34 |
|  | MOL002989 | 4-Hydroxyhomopterocarpin | 48.41 | 0.43 |
|  | MOL002990 | (6aR,11aR)-3,9,10-trimethoxy-6a,11a-dihydro-6H-benzofurano[3,2-c]chromen-4-ol | 66.86 | 0.53 |
|  | MOL002991 | (6aR,11aR)-3,9-dimethoxy-6a,11a-dihydro-6H-benzofurano[3,2-c]chromene-4,10-diol | 38.96 | 0.48 |
|  | MOL002996 | odoricarpin | 55.02 | 0.53 |
|  | MOL002997 | 3-(2-hydroxy-3,4-dimethoxyphenyl)-2H-chromen-7-ol | 86.18 | 0.27 |
|  | MOL002999 | Sativanone | 85.63 | 0.27 |
|  | MOL003000 | Stevein | 36.54 | 0.24 |
|  | MOL003001 | Vestitone | 52.83 | 0.24 |
|  | MOL003002 | violanone | 80.24 | 0.3 |
|  | MOL003003 | Xenognosin B | 72.71 | 0.24 |
|  | MOL000358 | beta-sitosterol | 36.91 | 0.75 |
|  | MOL000359 | sitosterol | 36.91 | 0.75 |
|  | MOL000380 | (6aR,11aR)-9,10-dimethoxy-6a,11a-dihydro-6H-benzofurano[3,2-c]chromen-3-ol | 64.26 | 0.42 |
|  | MOL000392 | formononetin | 69.67 | 0.21 |
| Juemingzi | MOL002268 | rhein | 47.07 | 0.28 |
|  | MOL002281 | Toralactone | 46.46 | 0.24 |
|  | MOL000449 | Stigmasterol | 43.83 | 0.76 |
|  | MOL000471 | aloe-emodin | 83.38 | 0.24 |
|  | MOL005043 | campest-5-en-3beta-ol | 37.58 | 0.71 |
|  | MOL006465 | Rubrofusarin-6-beta-gentiobioside | 40.12 | 0.67 |
|  | MOL006466 | Rubrofusarin | 45.55 | 0.24 |
|  | MOL006472 | Aurantio-obtusin | 31.55 | 0.37 |
|  | MOL006475 | Obtusin | 81.43 | 0.4 |
|  | MOL006481 | gluco-obtusifolin | 42.41 | 0.81 |
|  | MOL006482 | 9,10-dihydroxy-7-methoxy-3-methylene-4H-benzo[g]isochromen-1-one | 63.25 | 0.24 |
|  | MOL006486 | obtusin | 31.24 | 0.4 |
|  | MOL006489 | Quinizarin | 47.34 | 0.19 |
|  | MOL000953 | CLR | 37.87 | 0.68 |

**Table S2.** Active ingredients and associated targets.

| Chinese pinyin name | Mol ID | Latin name | Molecule Name | number of Degrees |
| --- | --- | --- | --- | --- |
| Huangqi | MOL000098 | Hedysarum Multijugum Maxim. | quercetin | 100 |
| Danshen | MOL000006 | Radix Salviae | luteolin | 44 |
| Baishao | MOL000422 | [Paeoniae Radix Alba](https://www.tcmsp-e.com/tcmspsearch.php?qr=Paeoniae%20Radix%20Alba&qsr=herb_en_name&token=654e89707431b6f423a4373243829a33) | kaempferol | 39 |
| Danshen | MOL007154 | Radix Salviae | tanshinone iia | 23 |
| Huangqi | MOL000392 | Hedysarum Multijugum Maxim. | formononetin | 18 |
| Jiangxiang | MOL002961 | Dalbergiae Odoriferae Lignum | (-)-Vestitol | 18 |
| Huangqi | MOL000354 | Hedysarum Multijugum Maxim. | isorhamnetin | 18 |
| Juemingzi | MOL000471 | Cassiae Semen | aloe-emodin | 17 |
| Jiangxiang | MOL002985 | Dalbergiae Odoriferae Lignum | isoduartin | 17 |
| Huangqi | MOL000378 | Hedysarum Multijugum Maxim. | 7-O-methylisomucronulatol | 17 |
| Danshen | MOL007093 | Radix Salviae | dan-shexinkum d | 16 |
| Danshen | MOL007100 | Radix Salviae | dihydrotanshinlactone | 16 |
| Jiangxiang | MOL002999 | Dalbergiae Odoriferae Lignum | Sativanone | 15 |
| Jiangxiang | MOL000358 | Dalbergiae Odoriferae Lignum | beta-sitosterol | 14 |
| Jiangxiang | MOL002565 | Dalbergiae Odoriferae Lignum | Medicarpin | 14 |
| Jiangxiang | MOL002959 | Dalbergiae Odoriferae Lignum | 3'-Methoxydaidzein | 14 |
| Jiangxiang | MOL002963 | Dalbergiae Odoriferae Lignum | 4',5',7-trimethyl-3-methoxyflavone | 14 |
| Jiangxiang | MOL002981 | Dalbergiae Odoriferae Lignum | Duartin | 14 |
| Jiangxiang | MOL003003 | Dalbergiae Odoriferae Lignum | Xenognosin B | 14 |
| Huangqi | MOL000417 | Hedysarum Multijugum Maxim. | Calycosin | 14 |
| Danshen | MOL007088 | Radix Salviae | cryptotanshinone | 14 |
| Danshen | MOL007108 | Radix Salviae | isocryptotanshi-none | 14 |
| Jiangxiang | MOL002941 | Dalbergiae Odoriferae Lignum | (3R)-3-(2,3-dihydroxy-4-methoxyphenyl)chroman-7,8-diol | 13 |
| Jiangxiang | MOL003001 | Dalbergiae Odoriferae Lignum | Vestitone | 13 |
| Jiangxiang | MOL002940 | Dalbergiae Odoriferae Lignum | (3R)-3-(2,3-dihydroxy-4-methoxyphenyl)-7-hydroxychroman-4-one | 12 |
| Jiangxiang | MOL002950 | Dalbergiae Odoriferae Lignum | (3R)-7,2',3'-trihydroxy-4-methoxyisoflavan | 12 |
| Jiangxiang | MOL002962 | Dalbergiae Odoriferae Lignum | (3S)-7-hydroxy-3-(2,3,4-trimethoxyphenyl)chroman-4-one | 12 |
| Danshen | MOL007049 | Radix Salviae | 4-methylenemiltirone | 12 |
| Danshen | MOL007119 | Radix Salviae | miltionone Ⅰ | 12 |
| Danshen | MOL007124 | Radix Salviae | neocryptotanshinone ii | 12 |
| Huangqi | MOL000380 | Hedysarum Multijugum Maxim. | (6aR,11aR)-9,10-dimethoxy-6a,11a-dihydro-6H-benzofurano[3,2-c]chromen-3-ol | 11 |
| Danshen | MOL007041 | Radix Salviae | 2-isopropyl-8-methylphenanthrene-3,4-dione | 11 |
| Danshen | MOL007094 | Radix Salviae | danshenspiroketallactone | 11 |
| Danshen | MOL007098 | Radix Salviae | deoxyneocryptotanshinone | 11 |
| Danshen | MOL007111 | Radix Salviae | Isotanshinone II | 11 |
| Jiangxiang | MOL002989 | Dalbergiae Odoriferae Lignum | 4-Hydroxyhomopterocarpin | 10 |
| Jiangxiang | MOL003002 | Dalbergiae Odoriferae Lignum | violanone | 10 |
| Huangqi | MOL000371 | Hedysarum Multijugum Maxim. | 3,9-di-O-methylnissolin | 10 |
| Danshen | MOL001601 | Radix Salviae | 1,2,5,6-tetrahydrotanshinone | 10 |
| Danshen | MOL007061 | Radix Salviae | Methylenetanshinquinone | 10 |
| Juemingzi | MOL006482 | Cassiae Semen | 9,10-dihydroxy-7-methoxy-3-methylene-4H-benzo[g]isochromen-1-one | 9 |
| Jiangxiang | MOL002938 | Dalbergiae Odoriferae Lignum | (3R)-4'-Methoxy-2',3,7-trihydroxyisoflavanone | 9 |
| Jiangxiang | MOL002990 | Dalbergiae Odoriferae Lignum | (6aR,11aR)-3,9,10-trimethoxy-6a,11a-dihydro-6H-benzofurano[3,2-c]chromen-4-ol | 9 |
| Jiangxiang | MOL002996 | Dalbergiae Odoriferae Lignum | odoricarpin | 9 |
| Huangqi | MOL000239 | Hedysarum Multijugum Maxim. | Jaranol | 9 |
| Guizhi | MOL004576 | Cinnamomi Ramulus | taxifolin | 9 |
| Danshen | MOL007050 | Radix Salviae | 2-(4-hydroxy-3-methoxyphenyl)-5-(3-hydroxypropyl)-7-methoxy-3-benzofurancarboxaldehyde | 9 |
| Danshen | MOL007059 | Radix Salviae | 3-beta-Hydroxymethyllenetanshiquinone | 9 |
| Danshen | MOL007105 | Radix Salviae | epidanshenspiroketallactone | 9 |
| Danshen | MOL007127 | Radix Salviae | 1-methyl-8,9-dihydro-7H-naphtho[5,6-g]benzofuran-6,10,11-trione | 9 |
| Juemingzi | MOL000449 | Cassiae Semen | Stigmasterol | 8 |
| Jiangxiang | MOL002966 | Dalbergiae Odoriferae Lignum | Dalbergin | 8 |
| Ganjiang | MOL002514 | [Zingiberis Rhizoma](https://www.tcmsp-e.com/tcmspsearch.php?qr=Zingiberis%20Rhizoma&qsr=herb_en_name&token=654e89707431b6f423a4373243829a33) | Sexangularetin | 8 |
| Danshen | MOL002651 | Radix Salviae | Dehydrotanshinone II A | 8 |
| Danshen | MOL007036 | Radix Salviae | 5,6-dihydroxy-7-isopropyl-1,1-dimethyl-2,3-dihydrophenanthren-4-one | 8 |
| Danshen | MOL007069 | Radix Salviae | przewaquinone c | 8 |
| Danshen | MOL007122 | Radix Salviae | Miltirone | 8 |
| Danshen | MOL007145 | Radix Salviae | salviolone | 8 |
| Jiangxiang | MOL000228 | Dalbergiae Odoriferae Lignum | (2R)-7-hydroxy-5-methoxy-2-phenylchroman-4-one | 7 |
| Jiangxiang | MOL002991 | Dalbergiae Odoriferae Lignum | (6aR,11aR)-3,9-dimethoxy-6a,11a-dihydro-6H-benzofurano[3,2-c]chromene-4,10-diol | 7 |
| Danshen | MOL007070 | Radix Salviae | (6S,7R)-6,7-dihydroxy-1,6-dimethyl-8,9-dihydro-7H-naphtho[8,7-g]benzofuran-10,11-dione | 7 |
| Danshen | MOL007081 | Radix Salviae | Danshenol B | 7 |
| Danshen | MOL007101 | Radix Salviae | dihydrotanshinoneⅠ | 7 |
| Danshen | MOL007125 | Radix Salviae | neocryptotanshinone | 7 |
| Danshen | MOL007130 | Radix Salviae | prolithospermic acid | 7 |
| Danshen | MOL007150 | Radix Salviae | (6S)-6-hydroxy-1-methyl-6-methylol-8,9-dihydro-7H-naphtho[8,7-g]benzofuran-10,11-quinone | 7 |
| Danshen | MOL007156 | Radix Salviae | tanshinone Ⅵ | 7 |
| Guizhi | MOL000492 | Cinnamomi Ramulus | (+)-catechin | 6 |
| Juemingzi | MOL002281 | Cassiae Semen | Toralactone | 6 |
| Jiangxiang | MOL001040 | Dalbergiae Odoriferae Lignum | (2R)-5,7-dihydroxy-2-(4-hydroxyphenyl)chroman-4-one | 6 |
| Jiangxiang | MOL001792 | Dalbergiae Odoriferae Lignum | DFV | 6 |
| Jiangxiang | MOL003000 | Dalbergiae Odoriferae Lignum | Stevein | 6 |
| Fuzi | MOL002392 | [Aconiti Lateralis Radix Praeparata](https://www.tcmsp-e.com/tcmspsearch.php?qr=Aconiti%20Lateralis%20Radix%20Praeparata&qsr=herb_en_name&token=654e89707431b6f423a4373243829a33) | Deltoin | 6 |
| Danshen | MOL002222 | Radix Salviae | sugiol | 6 |
| Danshen | MOL007045 | Radix Salviae | 3α-hydroxytanshinoneⅡa | 6 |
| Danshen | MOL007068 | Radix Salviae | Przewaquinone B | 6 |
| Danshen | MOL007079 | Radix Salviae | tanshinaldehyde | 6 |
| Danshen | MOL007085 | Radix Salviae | Salvilenone | 6 |
| Danshen | MOL007120 | Radix Salviae | miltionone Ⅱ | 6 |
| Danshen | MOL007132 | Radix Salviae | (2R)-3-(3,4-dihydroxyphenyl)-2-[(Z)-3-(3,4-dihydroxyphenyl)acryloyl]oxy-propionic acid | 6 |
| Danshen | MOL007143 | Radix Salviae | salvilenone Ⅰ | 6 |
| Danshen | MOL007151 | Radix Salviae | Tanshindiol B | 6 |
| Danshen | MOL007152 | Radix Salviae | Przewaquinone E | 6 |
| Danshen | MOL007155 | Radix Salviae | (6S)-6-(hydroxymethyl)-1,6-dimethyl-8,9-dihydro-7H-naphtho[8,7-g]benzofuran-10,11-dione | 6 |
| Baizhu | MOL000049 | [Atractylodes Macrocephala Koidz.](https://www.tcmsp-e.com/tcmspsearch.php?qr=Atractylodes%20Macrocephala%20Koidz.&qsr=herb_en_name&token=654e89707431b6f423a4373243829a33) | 3β-acetoxyatractylone | 6 |
| Jiangxiang | MOL002957 | Dalbergiae Odoriferae Lignum | 9-O-Methylcoumestrol | 5 |
| Huangqi | MOL000296 | Hedysarum Multijugum Maxim. | hederagenin | 5 |
| Danshen | MOL007058 | Radix Salviae | formyltanshinone | 5 |
| Jiangxiang | MOL002975 | Dalbergiae Odoriferae Lignum | butin | 4 |
| Huangqi | MOL000387 | Hedysarum Multijugum Maxim. | Bifendate | 4 |
| Huangqi | MOL000442 | Hedysarum Multijugum Maxim. | 1,7-Dihydroxy-3,9-dimethoxy pterocarpene | 4 |
| Guizhi | MOL000073 | Cinnamomi Ramulus | ent-Epicatechin | 4 |
| Fuzi | MOL002388 | [Aconiti Lateralis Radix Praeparata](https://www.tcmsp-e.com/tcmspsearch.php?qr=Aconiti%20Lateralis%20Radix%20Praeparata&qsr=herb_en_name&token=654e89707431b6f423a4373243829a33) | Delphin_qt | 4 |
| Danshen | MOL007064 | Radix Salviae | przewalskin b | 4 |
| Danshen | MOL007071 | Radix Salviae | przewaquinone f | 4 |
| Danshen | MOL007082 | Radix Salviae | Danshenol A | 4 |
| Danshen | MOL007107 | Radix Salviae | C09092 | 4 |
| Juemingzi | MOL002268 | Cassiae Semen | rhein | 3 |
| Juemingzi | MOL006486 | Cassiae Semen | obtusin | 3 |
| Juemingzi | MOL006489 | Cassiae Semen | Quinizarin | 3 |
| Guizhi | MOL001736 | Cinnamomi Ramulus | (-)-taxifolin | 3 |
| Fuzi | MOL002395 | [Aconiti Lateralis Radix Praeparata](https://www.tcmsp-e.com/tcmspsearch.php?qr=Aconiti%20Lateralis%20Radix%20Praeparata&qsr=herb_en_name&token=654e89707431b6f423a4373243829a33) | Deoxyandrographolide | 3 |
| Fuzi | MOL002398 | [Aconiti Lateralis Radix Praeparata](https://www.tcmsp-e.com/tcmspsearch.php?qr=Aconiti%20Lateralis%20Radix%20Praeparata&qsr=herb_en_name&token=654e89707431b6f423a4373243829a33) | Karanjin | 3 |
| Baishao | MOL001924 | [Paeoniae Radix Alba](https://www.tcmsp-e.com/tcmspsearch.php?qr=Paeoniae%20Radix%20Alba&qsr=herb_en_name&token=654e89707431b6f423a4373243829a33) | paeoniflorin | 3 |
| Juemingzi | MOL006475 | Cassiae Semen | Obtusin | 2 |
| Huangqi | MOL000433 | Hedysarum Multijugum Maxim. | FA | 2 |
| Ganjiang | MOL002501 | [Zingiberis Rhizoma](https://www.tcmsp-e.com/tcmspsearch.php?qr=Zingiberis%20Rhizoma&qsr=herb_en_name&token=654e89707431b6f423a4373243829a33) | [(1S)-3-[(E)-but-2-enyl]-2-methyl-4-oxo-1-cyclopent-2-enyl] (1R,3R)-3-[(E)-3-methoxy-2-methyl-3-oxoprop-1-enyl]-2,2-dimethylcyclopropane-1-carboxylate | 2 |
| Danshen | MOL000569 | Radix Salviae | digallate | 2 |
| Danshen | MOL007048 | Radix Salviae | (E)-3-[2-(3,4-dihydroxyphenyl)-7-hydroxy-benzofuran-4-yl]acrylic acid | 2 |
| Danshen | MOL007121 | Radix Salviae | miltipolone | 2 |
| Baizhu | MOL000072 | [Atractylodes Macrocephala Koidz.](https://www.tcmsp-e.com/tcmspsearch.php?qr=Atractylodes%20Macrocephala%20Koidz.&qsr=herb_en_name&token=654e89707431b6f423a4373243829a33) | 8β-ethoxy atractylenolide Ⅲ | 2 |
| Guizhi | MOL000359 | Cinnamomi Ramulus | sitosterol | 1 |
| Baizhu | MOL000033 | [Atractylodes Macrocephala Koidz.](https://www.tcmsp-e.com/tcmspsearch.php?qr=Atractylodes%20Macrocephala%20Koidz.&qsr=herb_en_name&token=654e89707431b6f423a4373243829a33) | (3S,8S,9S,10R,13R,14S,17R)-10,13-dimethyl-17-[(2R,5S)-5-propan-2-yloctan-2-yl]-2,3,4,7,8,9,11,12,14,15,16,17-dodecahydro-1H-cyclopenta[a]phenanthren-3-ol | 1 |
| Baishao | MOL000211 | [Paeoniae Radix Alba](https://www.tcmsp-e.com/tcmspsearch.php?qr=Paeoniae%20Radix%20Alba&qsr=herb_en_name&token=654e89707431b6f423a4373243829a33) | Mairin | 1 |
| Juemingzi | MOL005043 | Cassiae Semen | campest-5-en-3beta-ol | 1 |
| Juemingzi | MOL006472 | Cassiae Semen | Aurantio-obtusin | 1 |
| Juemingzi | MOL006481 | Cassiae Semen | gluco-obtusifolin | 1 |
| Juemingzi | MOL000953 | Cassiae Semen | CLR | 1 |
| Huangqi | MOL000379 | Hedysarum Multijugum Maxim. | 9,10-dimethoxypterocarpan-3-O-β-D-glucoside | 1 |
| Ganjiang | MOL002464 | [Zingiberis Rhizoma](https://www.tcmsp-e.com/tcmspsearch.php?qr=Zingiberis%20Rhizoma&qsr=herb_en_name&token=654e89707431b6f423a4373243829a33) | 1-Monolinolein | 1 |
| Danshen | MOL001659 | Radix Salviae | Poriferasterol | 1 |
| Danshen | MOL001771 | Radix Salviae | poriferast-5-en-3beta-ol | 1 |
| Danshen | MOL001942 | Radix Salviae | isoimperatorin | 1 |
| Danshen | MOL007063 | Radix Salviae | przewalskin a | 1 |
| Danshen | MOL007077 | Radix Salviae | sclareol | 1 |
| Danshen | MOL007141 | Radix Salviae | salvianolic acid g | 1 |
| Danshen | MOL007142 | Radix Salviae | salvianolic acid j | 1 |
| Baizhu | MOL000022 | [Atractylodes Macrocephala Koidz.](https://www.tcmsp-e.com/tcmspsearch.php?qr=Atractylodes%20Macrocephala%20Koidz.&qsr=herb_en_name&token=654e89707431b6f423a4373243829a33) | 14-acetyl-12-senecioyl-2E,8Z,10E-atractylentriol | 1 |
| Baishao | MOL001919 | [Paeoniae Radix Alba](https://www.tcmsp-e.com/tcmspsearch.php?qr=Paeoniae%20Radix%20Alba&qsr=herb_en_name&token=654e89707431b6f423a4373243829a33) | (3S,5R,8R,9R,10S,14S)-3,17-dihydroxy-4,4,8,10,14-pentamethyl-2,3,5,6,7,9-hexahydro-1H-cyclopenta[a]phenanthrene-15,16-dione | 1 |

**Table S3.** Topological scores of hub targets.

| Target | Betweenness | Closeness | Degree | Eigenvector | Local Average Connectivity(LAC) |
| --- | --- | --- | --- | --- | --- |
| TP53 | 3298.086062 | 0.111785095 | 42 | 0.32685557 | 7.619047619 |
| STAT3 | 2042.881969 | 0.111015491 | 30 | 0.2771779 | 7.533333333 |
| AKT1 | 1269.146738 | 0.11025641 | 29 | 0.253849715 | 6.689655172 |
| ESR1 | 723.0658082 | 0.109414758 | 22 | 0.216688037 | 7.636363636 |
